# Trastuzumab in the Adjuvant Treatment of HER2-Positive Early Breast Cancer Patients: A Meta-Analysis of Published Randomized Controlled Trials

**DOI:** 10.1371/journal.pone.0021030

**Published:** 2011-06-09

**Authors:** Wenjin Yin, Yiwei Jiang, Zhenzhou Shen, Zhimin Shao, Jinsong Lu

**Affiliations:** Department of Breast Surgery, Fudan University Shanghai Cancer Center, Department of Oncology, Shanghai Medical College, Fudan University, Shanghai, China; Yale Medical School, United States of America

## Abstract

**Background:**

Adjuvant trastuzumab therapy has yielded conflicting results for overall survival, concerns about central nervous system (CNS) metastasis, and questions about optimal schedule. Therefore, we carried out a meta-analysis to assess the benefits of concurrent or sequential trastuzumab with adjuvant chemotherapy for early breast cancer patients with HER2-positive tumors.

**Methods:**

Computerized and manual searches were performed to identify randomized clinical trials comparing adjuvant chemotherapy with or without trastuzumab in HER2-positive early breast cancer patients. Odds ratios were used to estimate the association between the addition of trastuzumab to adjuvant chemotherapy and various survival outcomes. The fixed-effects or random-effects model was used to combine data.

**Findings:**

With six eligible studies identified, this analysis demonstrated that patients with HER2-positive breast cancer derived benefit in disease-free survival, overall survival, locoregional recurrence and distant recurrence (all *P*<0.001) from the addition of trastuzumab to adjuvant chemotherapy, whereas trastuzumab did worse in CNS recurrence as compared to the control group (*P* = 0.018). Furthermore, concomitant use of trastuzumab significantly lowered the hazard of death (*P*<0.001) but bore a higher incidence of CNS recurrence (*P* = 0.010), while statistical significance failed to be discerned for either overall survival (*P* = 0.069) or CNS metastasis (*P* = 0.374) between the sequential and observation arms.

**Conclusion:**

This analysis verifies the efficacy of trastuzumab in the adjuvant setting. Additionally, our findings indirectly corroborate the superiority of concurrent trastuzumab to sequential use and also illuminate that prolonged survival is the possible reason for the higher incidence of CNS with trastuzumab versus observation.

## Introduction

Over recent years, breast cancer remains the most common cancer in women in western countries along with the sharp increase in its incidence rate in developing countries [1]. By contrast, the mortality rate has declined dramatically since the introduction of adjuvant systemic therapy, which is now used extensively for its established benefit in survival [2]. However, a considerable number of patients experience disease progression despite optimal postoperative treatments, which hints at a demand for novel agents against specific targets with greater efficacy and lesser toxicity [3].

Nowadays, an increasing number of targets have been yielded through various drug discovery efforts and some of these promising leads have moved beyond the stage of drug candidate, as exemplified by human epidermal growth factor receptor 2 (HER2) and trastuzumab. HER2 protein, encoded by the HER2/neu gene, belongs to a family of four homologous transmembrane receptors involved in tyrosine kinase-mediated regulation of normal breast tissue growth, differentiation, and survival. Overexpression of the HER2/neu protein (immunohistochemistry 3+), amplification of the HER2/neu gene (fluorescence *in situ* hybridization positive or chromogenic *in situ* hybridization positive), or both accounts for approximately 20–25% of invasive ductal carcinomas [4], [5], which is associated with aggressive clinical history in breast cancer patients. Trastuzumab, a humanized monoclonal antibody blocking the HER2/neu receptor, is one of the targeted approaches to reach the clinic for breast cancer. Initially, trastuzumab demonstrated significant activity in patients with HER2-positive metastatic breast cancer when combined with chemotherapy [6]. Such compelling evidence subsequently triggered the conduct of several large multicenter randomized controlled clinical trials assessing sequential or concurrent trastuzumab with chemotherapy in the adjuvant setting. These impressive results have also unequivocally revealed the striking effect of adjuvant trastuzumab on the improvement of HER2-positive breast cancer prognosis.

Unfortunately, many problems are still in the air when it came to trastuzumab administration after surgery. Firstly, the definitely significant efficacy of trastuzumab was merely confined to the primary endpoint, disease-free survival (DFS) [7]–[12], while conflicting results were reported with regard to overall survival. In the HERA trial, the 2-year follow-up report revealed that adjuvant treatment with trastuzumab for 1 year lowered risk of death by 34% as compared to the observation group, which corresponded to an absolute overall survival benefit of 2.7% at 36 months with a statistically significant difference (*P* = 0.0115) [11]. However, after a median follow-up of 4 years, the absolute benefit in overall survival declined to 1.6% at 48 months and statistical significance was not observed between the two groups [7], [12]. Some investigators argued that crossover to trastuzumab of patients originally allocated to the observation arm might disrupt and bias the intention-to-treat (ITT) comparison between the 1-year trastuzumab and observation arms [7], [12], but others took issue with this line of thought since the findings from the PACS 04 and FinHer studies revealed that adjuvant trastuzumab did not affect overall survival despite the lack of crossover [13]–[15]. Secondly, many oncologists reported frequent central nervous system (CNS) metastasis during trastuzumab therapy in several clinical trials [9], [11], [13]–[15], which has raised burning concerns over the association of trastuzumab use with the development of CNS recurrence. Thirdly, there is growing attention toward how to optimize the combination of trastuzumab with adjuvant chemotherapy. Updated results of the NCCTG N9831 study demonstrated a strong trend for an absolute benefit in DFS of 4.4% at 5 years with concomitant trastuzumab relative to sequential administration (*P* = 0.019). Although this did not cross the boundary for statistical significance (preset at 0.00116), investigators recommended incorporation of adjuvant trastuzumab concurrent with the taxane portion of chemotherapy [8]. Nevertheless, currently available data are still not adequate to judge which schedule is superior in efficacy, prompting further investigation for additional evidence. Last but not least, relevant analyses from several large randomized clinical trials have been much updated recently, which makes it obliged to obtain the timely insight into a paradigm shift in how we deal with HER2-positive breast cancer patients in the adjuvant setting.

On account of the above points, we carried out a meta-analysis to assess the prognostic effect of trastuzumab administration after surgery in early breast cancer patients with HER2-positive tumors so as to get a clear picture of the benefits it offers, providing the implications for the appropriate use of trastuzumab in clinical practice.

## Methods

### Publication search

The selection of publications for inclusion was performed independently by two of the authors (Wenjin Yin and Yiwei Jiang), with the last search on September 20, 2010. A computerized search was performed through the PubMed database (from 1966 to the present), the online proceedings of the American Society of Clinical Oncology (ASCO) Annual Meetings (years 1992–2010), the online proceedings of the San Antonio Breast Cancer Symposium (years 2004–2009), and the CD proceedings of the International St. Gallen Breast Cancer Conference (years 2003–2009), using the following search keywords: “trastuzumab” or “Hercetpin”, “adjuvant” or “postoperative”, and “breast cancer”. Manual searches were done by reviewing the reference lists of retrieved studies, textbooks and review articles to identify additional potentially eligible studies. The language of publication was restricted to English.

### Eligibility criteria

This analysis included all randomized controlled trials that evaluated adjuvant chemotherapy with (concurrent or sequential) or without the administration of trastuzumab at any dose and for any duration among patients with HER2-positive invasive breast cancer in the adjuvant setting. Interim analyses of trials or studies presented in abstract form were considered eligible only if they elucidated the latest available data on at least one of the endpoints in this analysis. In case of multiple reports on the same trial arm, the one with the longest follow-up interval was applied for the calculations. Surgical modality, together with schemes of adjuvant chemotherapy, radiotherapy and endocrine therapy, was not considered for study selection. Trials were required not to test trastuzumab as neoadjuvant or salvage treatment, as well as not to evaluate biological response modifiers or targeted agents other than trastuzumab.

### Data extraction

The following information was extracted from each publication: journal name, year of publication, first author, follow-up period, number of patients analyzed per arm, chemotherapy and trastuzumab regimens, and number of endpoint events per arm. In the present analysis, two of the authors (Wenjin Yin and Yiwei Jiang) independently extracted the information from each eligible publication. All the relevant data were further reviewed by a third investigator (Jinsong Lu) to reach consensus. No authors of the original publications were contacted for verification or clarification of their data.

### Study endpoint

In this meta-analysis, the primary outcome was DFS, defined as time from randomization to the first occurrence of disease progression or death from any cause without documentation of a cancer-related event. Secondary outcomes included overall survival (death from any cause), time to locoregional recurrence, time to distant recurrence, time to contralateral breast cancer, and time to CNS recurrence.

### Statistical analysis

All analyses were conducted according to the ITT principle. Odds ratios (ORs) with their 95% confidence intervals (CIs) were calculated from the number of outcome events per arm to estimate the association between the addition of trastuzumab to adjuvant chemotherapy and various survival outcomes. The heterogeneity of the study results was assessed by Cochran chi-square Q statistics and I-square test, which determined the use of either fixed-effects (Mantel-Haenszel method) or random-effects (DerSimonian and Laird method) model. Heterogeneity was considered as either a *P*-value<0.05 or I-square>50% [16].

Funnel plots, contour-enhanced funnel plots, and Egger's test were used to evaluate the possible publication bias regarding each study outcome. The fail-safe number was employed to estimate the minimum number of “missing” studies averaging null results or no effect that would be needed to overturn the conclusion reached in the meta-analysis. Sensitivity analyses were conducted to assess the influence of specific studies on the combined effect.

All tests were two-sided and *P*<0.05 was considered significant. All statistical analyses were performed with Stata statistical software package (release 10.0; Stata Corporation, College Station, Texas, USA) and Comprehensive Meta-analysis software program (Version 2.2.034, Biostat, Englewood NJ).

## Results

### Description of eligible studies

Based on the search strategy, six eligible studies were identified. According to the timing of trastuzumab initiation with respect to chemotherapy, all the trastuzumab arms from these trials were categorized into the concurrent or sequential subgroup. The study characteristics are summarized in Table 1.

**Table 1:** Characteristics of eligible studies included in the meta-analysis.

Study	Median follow-up (months)	Treatment regimen per arm	Timing of trastuzumab initiation with respect to chemotherapy	Duration of trastuzumab administration (weeks)	Numuber of patients
BCIRG 006 [10]	65	AC→D	-	-	1073
		AC→D+T→T	concurrent	52	1074
		D+Carbo+T→T	concurrent	52	1075
FinHer [13], [14]	62	D/V→FEC	-	-	116
		D/V+T→FEC	concurrent	9	115
HERA [7], [11], [12]	48.4	CT±RT→observation	-	-	1698
		CT±RT→T×1 year	sequential	52	1703
		CT±RT→T×2 years	sequential	104	1701
NCCTG N9831 [8], [9]	66	AC→P	-	-	1087
		AC→P+T→T	concurrent	52	949
		AC→P→T	sequential	52	1097
NSABP B31 [9]	28.8	AC→P	-	-	872
		AC→P+T→T	concurrent	52	864
PACS 04 [15]	47	FEC/ED±RT	-	-	268
		FEC/ED±RT→T	sequential	52	260

Abbreviations: BCIRG, Breast Cancer International Research Group; AC, doxorubicin and cyclophosphamide; D, docetaxel; T, trastuzumab; Carbo, carboplatin; FinHer, Finland Herceptin® study; V, vinorelbine; FEC, fluorouracil, epirubicin and cyclophosphamide; HERA, Herceptin® Adjuvant trial; CT, chemotherapy; RT, radiotherapy; NCCTG, North Central Cancer Treatment Group; P, paclitaxel; NSABP, National Surgical Adjuvant Breast and Bowel Project; PACS, French Protocol Adjuvant dans le Cancer du Sein; ED, epirubicin and docetaxel.

From the HERA study, the efficacy of trastuzumab for 2 years has not been reported, and therefore, this arm was excluded in this analysis [7], [11], [12]. Additionally, the latest analysis of the NCCTG N9831 study provided the results on the differences in DFS and OS between the concurrent and sequential arm rather than those between the concurrent and control arm [8]. By this token, the concurrent arm was excluded from the meta-analysis.

As to the FinHer trial, only the data of HER2-positive patients was extracted since it was a 2×2 study, where all the participants were randomly assigned to receive three cycles of vinorelbine or docetaxel, both followed by three cycles of fluorouracil, epirubicin and cyclophosphamide (FEC), and then HER2-positive women were further randomized to either trastuzumab or observation [13], [14].

When it came to the BCIRG 006 trial, controversies arose over whether to incorporate the non-anthracycline, carboplatin-based arm or not for the meta-analysis. Some investigators removed it for lack of an appropriate unconfounded control [17], [18]. However, there is no denying the fact that the addition of trastuzumab to docetaxel and carboplatin (DCarboT) was at least partially responsible for its superiority in efficacy over the anthracycline-based control arm on the basis of the available data. The exclusion of the DCarboT arm might sacrifice valuable information. For the sake of objective appraisal, the meta-analysis was conducted respectively with or without subsuming the DCarboT arm.

It is noteworthy that as for site-specific recurrence there is a distinction between events at any time and first events in this meta-analysis. If both were available (only found in the PACS 04 trial [15] concerning contralateral breast cancer), the former was preferred to the latter; otherwise, first events served as a substitute for events at any time.

### Overall effect of trastuzumab on primary outcome

There was significant between-study heterogeneity (Table S1) in the ORs for DFS (including the DCarboT arm of the BCIRG 006 trial, abbreviated to “DCarboT”; heterogeneity chi-squared = 14.32, I-squared = 58.1%, *P* = 0.026), so the random-effects model was used to analyze the data and demonstrated a DFS benefit in favor of trastuzumab combined with adjuvant chemotherapy as compared to chemotherapy alone (DCarboT; OR = 0.69, 95% CI 0.59–0.80, *P*<0.001; Figure S1). The similar result was yielded by the exclusion of the DCarboT arm in the BCIRG 006 trial (heterogeneity chi-squared = 12.59, I-squared = 60.3%, *P* = 0.027; OR = 0.67, 95% CI 0.56–0.79, *P*<0.001; Figure S2).

### Overall effects of trastuzumab on secondary outcomes

In terms of the secondary outcomes, there was no between-study heterogeneity (Table S1) in the ORs for overall survival (DCarboT; heterogeneity chi-squared = 6.13, I-squared = 18.5%, *P* = 0.293), time to locoregional recurrence (heterogeneity chi-squared = 2.87, I-squared = 0.0%, *P* = 0.412), time to distant recurrence (DCarboT; heterogeneity chi-squared = 8.30, I-squared = 39.7%, *P* = 0.141), time to contralateral breast cancer (heterogeneity chi-squared = 2.99, I-squared = 0.0%, *P* = 0.393), and time to CNS recurrence (heterogeneity chi-squared = 2.61, I-squared = 0.0%, *P* = 0.456).

**Figure 1:**
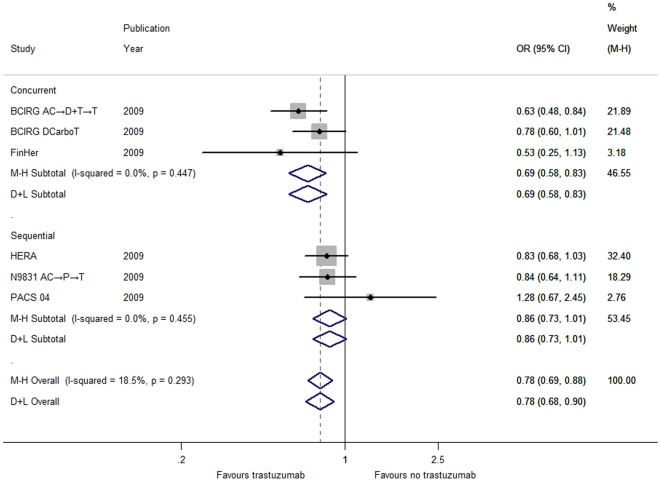
Forest plots of OR for the association between trastuzumab administration and overall survival by the timing of trastuzumab initiation with respect to chemotherapy (including the DCarboT arm of the BCIRG 006 trial). The size of the square box is proportional to the weight that each study contributes in the meta-analysis. The overall estimate and confidence interval are marked by a diamond. Symbols on the right of the solid line indicate OR>1 and symbols on the left of the solid line indicate OR<1. Abbreviations: M-H = Mantel-Haenszel (fixed-effects method); D+L = DerSimonian and Laird (random-effects method).

**Figure 2:**
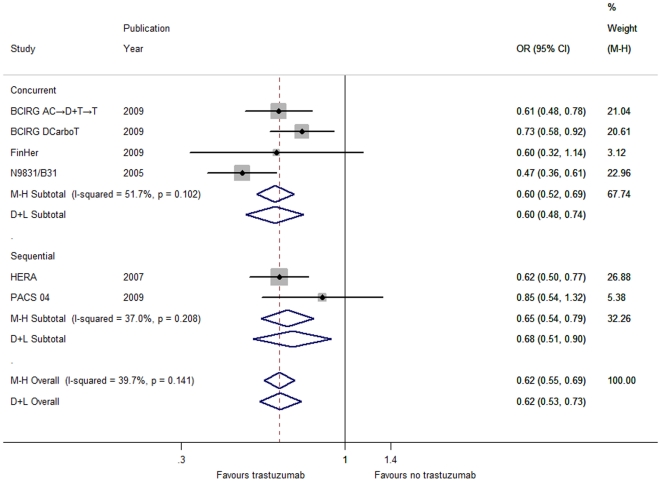
Forest plots of OR for the association between trastuzumab administration and distant recurrence by the timing of trastuzumab initiation with respect to chemotherapy (including the DCarboT arm of the BCIRG 006 trial). The size of the square box is proportional to the weight that each study contributes in the meta-analysis. The overall estimate and confidence interval are marked by a diamond. Symbols on the right of the solid line indicate OR>1 and symbols on the left of the solid line indicate OR<1. Abbreviations: M-H = Mantel-Haenszel (fixed-effects method); D+L = DerSimonian and Laird (random-effects method).

Through the fixed-effects model, we found that the addition of trastuzumab to adjuvant chemotherapy elicited a considerable reduction in risk (Table S1) respectively for death from any cause (DCarboT; OR = 0.78, 95% CI 0.69–0.88, *P*<0.001; Figure 1), locoregional recurrence (OR = 0.53, 95% CI 0.44–0.65, *P*<0.001; Figure S4) and distant recurrence (DCarboT; OR = 0.62, 95% CI 0.55–0.69, *P*<0.001; Figure 2) rather than for contralateral breast cancer (OR = 1.11, 95% CI 0.61–2.01, *P* = 0.737; Figure S6). Furthermore, the exclusion of the DCarboT arm failed to influence the overall results for both overall survival and time to distant recurrence (Figure S3 and Figure S5).

**Figure 3:**
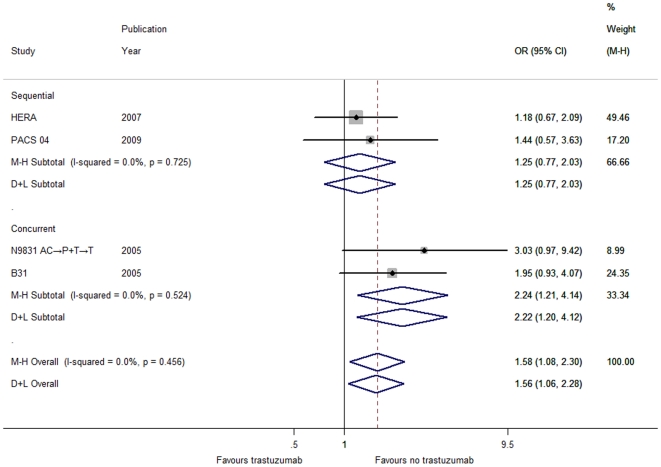
Forest plot of OR for the association between trastuzumab administration and CNS recurrence by the timing of trastuzumab initiation with respect to chemotherapy. The size of the square box is proportional to the weight that each study contributes in the meta-analysis. The overall estimate and confidence interval are marked by a diamond. Symbols on the right of the solid line indicate OR>1 and symbols on the left of the solid line indicate OR<1. Abbreviations: M-H = Mantel-Haenszel (fixed-effects method); D+L = DerSimonian and Laird (random-effects method).

Unexpectedly, a higher percentage of CNS recurrence was observed in patients receiving trastuzumab than that in the observation counterparts (OR = 1.58, 95% CI 1.08–2.30, *P* = 0.018; Table S1 and Figure 3).

### Effect of trastuzumab in subgroup by axillary lymph node (ALN) status

According to the number of ALN involvement, patients were divided into three subgroups as follows: negative (no ALN involved), 1–3 positive nodes and ≥4 positive nodes. Compared to chemotherapy alone, all the subgroups derived a greater benefit in DFS from trastuzumab in combination with chemotherapy no matter whether or not the DCarboT arm was included (Figure S7 and Figure S8).

### Effect of trastuzumab in subgroup by the timing of trastuzumab initiation with respect to chemotherapy

According to the timing of trastuzumab initiation with respect to chemotherapy, patients were categorized into the concurrent or sequential group. In terms of DFS (Figure S1), time to locoregional recurrence (Figure S4), time to distant recurrence (Figure 2) and time to contralateral breast cancer (Figure S6), the effect for each individual group was consistent with the overall pooled results (Table S1). Thereinto, whether the DCarboT arm was included or not did not influence the benefit trend (Table S1) in DFS (Figure S2) and time to distant recurrence (Figure S5). Interestingly, the effect of trastuzumab on overall survival and time to CNS recurrence differed widely between the two groups (Table S1). Concomitant use of trastuzumab significantly lowered the risk of mortality (Figure 1) but bore a higher incidence of CNS recurrence (Figure 3), while statistical significance failed to be discerned for both overall survival and CNS recurrence in the sequential group despite its similar direction in line with the aggregated data (Figure 1 and Figure 3).

### Publication bias and sensitivity analysis

Either graphical inspection for funnel plots and contour-enhanced funnel plots or quantitative evaluation from Egger's test indicated the absence of publication bias in DFS (DCarboT; t = −0.24, *P* = 0.821; Figure S9), overall survival (DCarboT; t = 0.03, *P* = 0.977), time to locoregional recurrence (t = 1.67, *P* = 0.237), time to distant recurrence (DCarboT; t = 0.41, *P* = 0.701), time to contralateral breast cancer (t = 1.31, *P* = 0.319) and time to CNS recurrence (t = 1.86, *P* = 0.204). What's more important, the conclusions above on DFS, overall survival and time to distant recurrence were not substantially altered by the exclusion of the DCarboT arm (figures and data not shown). The fail-safe number of missing studies for DFS that would bring the *P*-value to >0.05 was 124 and 100 respectively for the analysis with or without including the DCarboT arm. Similar results were yielded in terms of overall survival, time to locoregional recurrence, time to distant recurrence, time to contralateral breast cancer and time to CNS recurrence (figures and data not shown).

**Figure 4:**
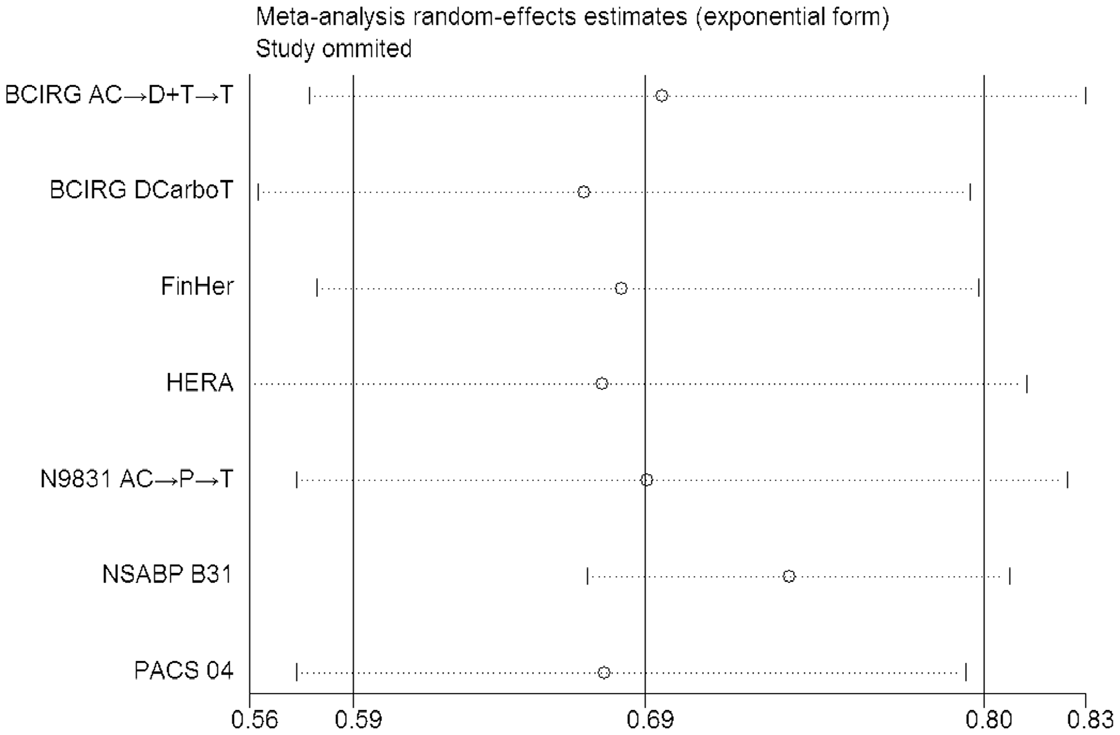
Sensitivity analyses for the influence of individual studies on the summary OR for the association between trastuzumab administration and DFS (including the DCarboT arm of the BCIRG 006 trial). The vertical midline indicates the overall OR and the lateral vertical lines indicate its 95% CI. Every hollow round indicates the pooled OR when the left study is omitted in this meta-analysis. The two ends of every dotted line represent the respective 95% CI.

The sensitivity analyses clarified that no individual study affected the overall OR for DFS regardless of whether the DCarboT arm was included or not, since omission of any single study made no material difference (Figure 4 and Figure S10). The same conclusions were drawn with respect of overall survival, time to locoregional recurrence, time to distant recurrence, time to contralateral breast cancer and time to CNS recurrence (figures not shown).

## Discussion

This analysis is, to the best of our knowledge, the largest and latest meta-analysis focusing on the administration of trastuzumab in the adjuvant setting, which incorporated six major randomized clinical trials with the longest follow-up interval [7]–[11], [13]–[15]. As an indispensable component of adjuvant systemic therapy for HER2-positive early breast cancer patients, trastuzumab has brought about a considerable decline in the risk of recurrence. In consistence with previous analyses [17], [18], we demonstrated the beneficial effect of trastuzumab in terms of DFS and time to distant recurrence. Furthermore, this meta-analysis was the first to provide substantial evidence for the risk reduction of trastuzumab in locoregional recurrence. Additionally, the effect of trastuzumab on overall survival requires consideration of selective crossover in the HERA trial for objective evaluation. In the observation group, 65% patients who were alive and disease free on 16 May 2005 crossed over and received trastuzumab [7], [12], which led to a dilution of treatment efficacy in the ITT analysis. Nevertheless, the overall pooled OR for all the studies included in this analysis exhibited a remarkable improvement of overall survival in favor of trastuzumab. Thus, these data served as a keystone for clinical practice toward treating HER2-positive early breast cancer patient with adjuvant trastuzumab.

Another issue merits special attention in this report that there seemed to be a higher rate of CNS recurrence in patients receiving adjuvant trastuzumab [17], [18]. What brings about this trend still remains unclear and open to investigation. CNS recurrence is, as a rule, preceded by metastases to other distant organs such as the lung, liver or bone since its late occurrence in the natural history of metastatic breast cancer [19]–[22]. Therefore, a nonexclusive hypothesis posits that better control of extracranial diseases with trastuzumab extended the periods of survival to such a degree as to display an increased propensity for CNS metastasis [23], [24]. As a result, CNS recurrence will in all likelihood become more prevalent, which prompts further study on how to prevent its development and tailor its treatment. Encouragingly, several case reports have clarified the successful treatment of meningeal carcinomatosis from breast cancer *via* intrathecal administration of trastuzumab [24]–[27].

Nowadays, much importance has also been attached on the timing of trastuzumab initiation with respect to chemotherapy. Among the six largest randomized trials included in this analysis, the N9831 study was the only one to directly compare the concurrent and sequential use of trastuzumab. The updated results ascertained a strong trend for patients with HER2-positive tumors to derive more benefit in DFS from concomitant trastuzumab rather than from sequential schedule [8]. In contrast, some investigators argue that no significant difference in overall survival was achieved between the two arms despite relative superiority of concurrent use [28]. Besides, the concurrent arm was temporarily closed due to its higher frequency of cardiac events than the sequential and observation peers. For these reasons, they maintain that it is still far too early to challenge the sequential administration of trastuzumab in view of both efficacy and safety [28]. Under such circumstances, it is most urgent for further probe into what schedule is the better between the concurrent and sequential use of trastuzumab. Interestingly, the present analysis first illustrated a significant effect of either concurrent or sequential trastuzumab on the improvement of DFS and extracranial recurrence when compared to the observation arm respectively, which had not been previously mentioned [17], [18]. Moreover, we bring it to light for the first time that patients receiving concomitant trastuzumab experienced a considerable reduction in mortality risk but a higher incidence of CNS recurrence relative to those without any trastuzumab treatment, while trastuzumab administration after completion of adjuvant chemotherapy/radiotherapy seemed not to notably ameliorate the overall survival and influence the rate of CNS metastasis. This intriguing phenomenon further suggests better efficacy of concurrent schedule than sequential use of trastuzumab. On the other hand, our findings first provide indirect evidence for the hypothesis just as mentioned above that superiority of concurrent schedule in preventing non-CNS recurrence contributes to a relatively extended life span so that metastatic propensity to CNS, usually occurring late in the course of the disease, is augmented as a natural consequence. Nonetheless, our findings are only for hypothesis generation and need to be confirmed through well-designed and well-executed randomized clinical trials.

Additionally, we demonstrated unfortunately in the present analysis that trastuzumab had no bearing on the risk reduction of contralateral breast cancer. But curiously enough, adjuvant endocrine therapy including tamoxifen and aromatase inhibitors brought about a definite and highly significant decrease in the incidence of contralateral breast cancer for women with estrogen receptor (ER)-positive or ER-unknown disease [2], [29]–[31]. As far as we know, this marked divergence had never been described before; nor had its underlying mechanism been elucidated. Whether it is attributed to the dissimilar function in carcinogenesis or metastasis between ER and HER2 still remains suspended and requires intensive study.

In conclusion, this analysis provides the latest and overwhelming evidence for the outstanding efficacy of adjuvant trastuzumab administration in reducing the risk of extracranial recurrence as well as prolonging the DFS and overall survival. More importantly, our findings first corroborate that concurrent trastuzumab outweighs sequential use as concerns overall survival advantage. Besides, we further certify the postulation that the rising level of CNS recurrence actually accompanies the life extension by treatment with trastuzumab vs. observation. Current fashion of administration might insufficiently maximize the prophylactic function of trastuzumab on CNS metastasis in the adjuvant setting rather than increase its incidence. We hypothesize that the combination of trastuzumab with other targeted agents easy to penetrate through BBB, such as lapatinib, is likely to suppress CNS recurrence, which will be partially explained by the Adjuvant Lapatinib And/Or Trastuzumab Treatment Optimisation (ALTTO) trial in the near future [32]. Therefore, it is fallacious to reject trastuzumab in the adjuvant setting out of concerns for CNS metastasis. On the contrary, efforts should be made to optimize the adjuvant trastuzumab therapy considering the poor prognosis of HER2-positive breast cancer patients, which stimulates the closer collaboration of basic, translational and clinical research for major breakthroughs.

## Supporting Information

Figure S1 **Forest plots of OR for the association between trastuzumab administration and DFS by the timing of trastuzumab initiation with respect to chemotherapy (including the DCarboT arm of the BCIRG 006 trial).** The size of the square box is proportional to the weight that each study contributes in the meta-analysis. The overall estimate and confidence interval are marked by a diamond. Symbols on the right of the solid line indicate OR>1 and symbols on the left of the solid line indicate OR<1. Abbreviations: M-H = Mantel-Haenszel (fixed-effects method); D+L = DerSimonian and Laird (random-effects method).
(TIF)

Figure S2 **Forest plots of OR for the association between trastuzumab administration and DFS by the timing of trastuzumab initiation with respect to chemotherapy without including the DCarboT arm of the BCIRG 006 trial.** The size of the square box is proportional to the weight that each study contributes in the meta-analysis. The overall estimate and confidence interval are marked by a diamond. Symbols on the right of the solid line indicate OR>1 and symbols on the left of the solid line indicate OR<1. Abbreviations: M-H = Mantel-Haenszel (fixed-effects method); D+L = DerSimonian and Laird (random-effects method).
(TIF)

Figure S3 **Forest plots of OR for the association between trastuzumab administration and overall survival by the timing of trastuzumab initiation with respect to chemotherapy without including the DCarboT arm of the BCIRG 006 trial.** The size of the square box is proportional to the weight that each study contributes in the meta-analysis. The overall estimate and confidence interval are marked by a diamond. Symbols on the right of the solid line indicate OR>1 and symbols on the left of the solid line indicate OR<1. Abbreviations: M-H = Mantel-Haenszel (fixed-effects method); D+L = DerSimonian and Laird (random-effects method).
(TIF)

Figure S4 **Forest plot of OR for the association between trastuzumab administration and locoregional recurrence by the timing of trastuzumab initiation with respect to chemotherapy.** The size of the square box is proportional to the weight that each study contributes in the meta-analysis. The overall estimate and confidence interval are marked by a diamond. Symbols on the right of the solid line indicate OR>1 and symbols on the left of the solid line indicate OR<1. Abbreviations: M-H = Mantel-Haenszel (fixed-effects method); D+L = DerSimonian and Laird (random-effects method).
(TIF)

Figure S5 **Forest plots of OR for the association between trastuzumab administration and distant recurrence by the timing of trastuzumab initiation with respect to chemotherapy without including the DCarboT arm of the BCIRG 006 trial.** The size of the square box is proportional to the weight that each study contributes in the meta-analysis. The overall estimate and confidence interval are marked by a diamond. Symbols on the right of the solid line indicate OR>1 and symbols on the left of the solid line indicate OR<1. Abbreviations: M-H = Mantel-Haenszel (fixed-effects method); D+L = DerSimonian and Laird (random-effects method).
(TIF)

Figure S6 **Forest plot of OR for the association between trastuzumab administration and contralateral breast cancer by the timing of trastuzumab initiation with respect to chemotherapy.** The size of the square box is proportional to the weight that each study contributes in the meta-analysis. The overall estimate and confidence interval are marked by a diamond. Symbols on the right of the solid line indicate OR>1 and symbols on the left of the solid line indicate OR<1. Abbreviations: M-H = Mantel-Haenszel (fixed-effects method); D+L = DerSimonian and Laird (random-effects method).
(TIF)

Figure S7 **Forest plots of OR for the association between trastuzumab administration and DFS by axillary lymph node status (including the DCarboT arm of the BCIRG 006 trial).** The size of the square box is proportional to the weight that each study contributes in the meta-analysis. The overall estimate and confidence interval are marked by a diamond. Symbols on the right of the solid line indicate OR>1 and symbols on the left of the solid line indicate OR<1. Abbreviations: M-H = Mantel-Haenszel (fixed-effects method); D+L = DerSimonian and Laird (random-effects method).
(TIF)

Figure S8 **Forest plots of OR for the association between trastuzumab administration and DFS by axillary lymph node status without including the DCarboT arm of the BCIRG 006 trial.** The size of the square box is proportional to the weight that each study contributes in the meta-analysis. The overall estimate and confidence interval are marked by a diamond. Symbols on the right of the solid line indicate OR>1 and symbols on the left of the solid line indicate OR<1. Abbreviations: M-H = Mantel-Haenszel (fixed-effects method); D+L = DerSimonian and Laird (random-effects method).
(TIF)

Figure S9 **Funnel plot for publication bias in DFS with the analysis including the DCarboT arm of the BCIRG 006 trial.** The red line indicates the fitted line corresponding to the Egger's regression test for funnel plot asymmetry.
(TIF)

Figure S10 **Sensitivity analyses for the influence of individual studies on the summary OR for the association between trastuzumab administration and DFS without including the DCarboT arm of the BCIRG 006 trial.** The vertical midline indicates the overall OR and the lateral vertical lines indicate its 95% CI. Every hollow round indicates the pooled OR when the left study is omitted in this meta-analysis. The two ends of every dotted line represent the respective 95% CI.
(TIF)

Table S1 **Effect of trastuzumab in subgroup by the timing of trastuzumab initiation with respect to adjuvant chemotherapy.**
(DOC)
