# Supplementary figures and images for: Trastuzumab in the Adjuvant Treatment of HER2-Positive Early Breast Cancer Patients: A Meta-Analysis of Published Randomized Controlled Trials
Source: PLoS One. 2011 Jun 9;6(6):e21030. doi: 10.1371/journal.pone.0021030 (PMC3111470; doi:10.1371/journal.pone.0021030)

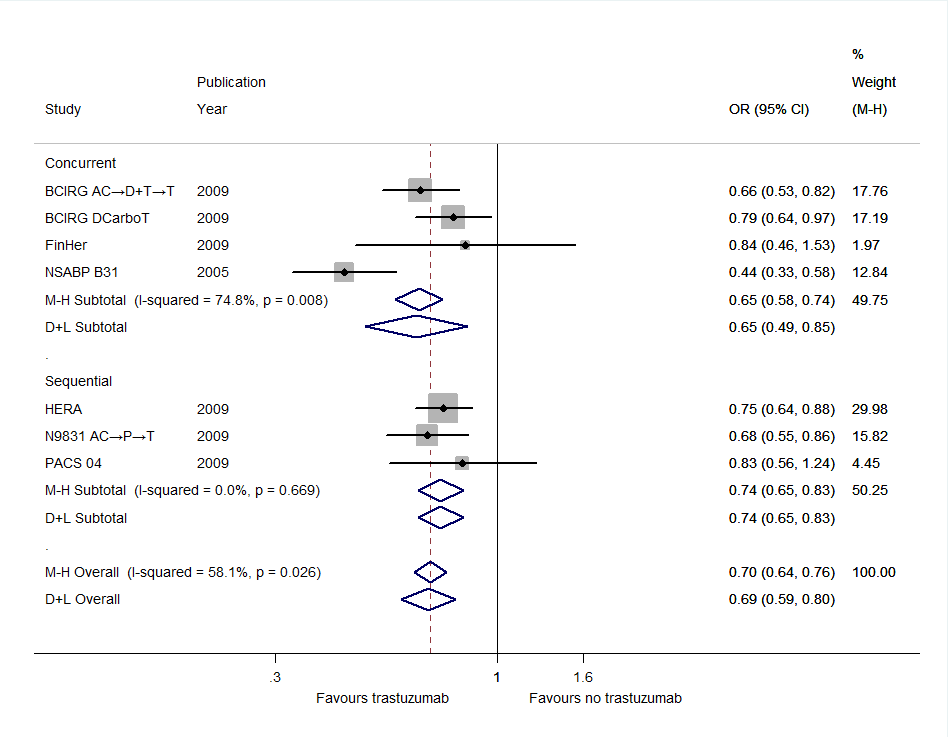

Supplement: Figure S1 — Forest plots of OR for the association between trastuzumab administration and DFS by the timing of trastuzumab initiation with respect to chemotherapy (including the DCarboT arm of the BCIRG 006 trial). The size of the square box is proportional to the weight that each study contributes in the meta-analysis. The overall estimate and confidence interval are marked by a diamond. Symbols on the right of the solid line indicate OR>1 and symbols on the left of the solid line indicate OR<1. Abbreviations: M-H = Mantel-Haenszel (fixed-effects method); D+L = DerSimonian and Laird (random-effects method). (TIF) [file pone.0021030.s001.tif]

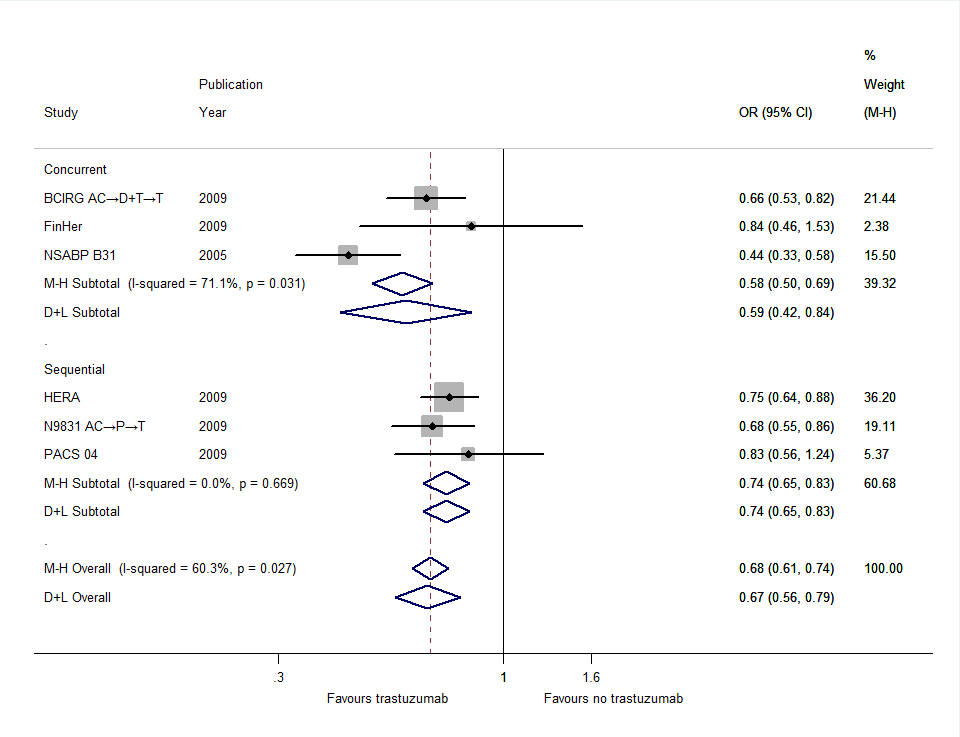

Supplement: Figure S2 — Forest plots of OR for the association between trastuzumab administration and DFS by the timing of trastuzumab initiation with respect to chemotherapy without including the DCarboT arm of the BCIRG 006 trial. The size of the square box is proportional to the weight that each study contributes in the meta-analysis. The overall estimate and confidence interval are marked by a diamond. Symbols on the right of the solid line indicate OR>1 and symbols on the left of the solid line indicate OR<1. Abbreviations: M-H = Mantel-Haenszel (fixed-effects method); D+L = DerSimonian and Laird (random-effects method). (TIF) [file pone.0021030.s002.tif]

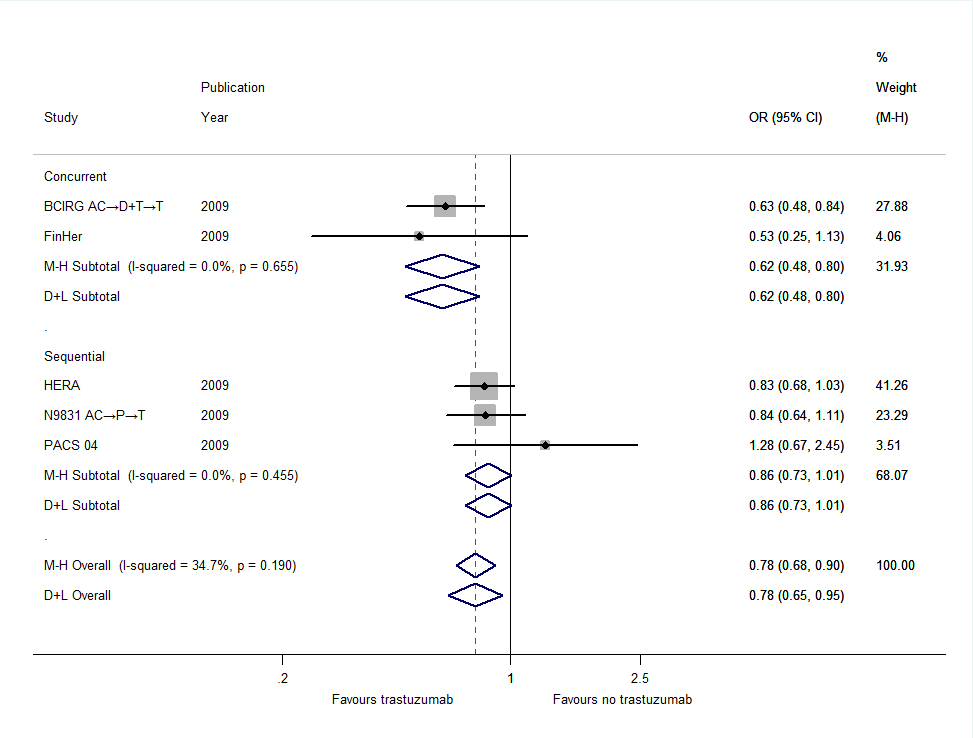

Supplement: Figure S3 — Forest plots of OR for the association between trastuzumab administration and overall survival by the timing of trastuzumab initiation with respect to chemotherapy without including the DCarboT arm of the BCIRG 006 trial. The size of the square box is proportional to the weight that each study contributes in the meta-analysis. The overall estimate and confidence interval are marked by a diamond. Symbols on the right of the solid line indicate OR>1 and symbols on the left of the solid line indicate OR<1. Abbreviations: M-H = Mantel-Haenszel (fixed-effects method); D+L = DerSimonian and Laird (random-effects method). (TIF) [file pone.0021030.s003.tif]

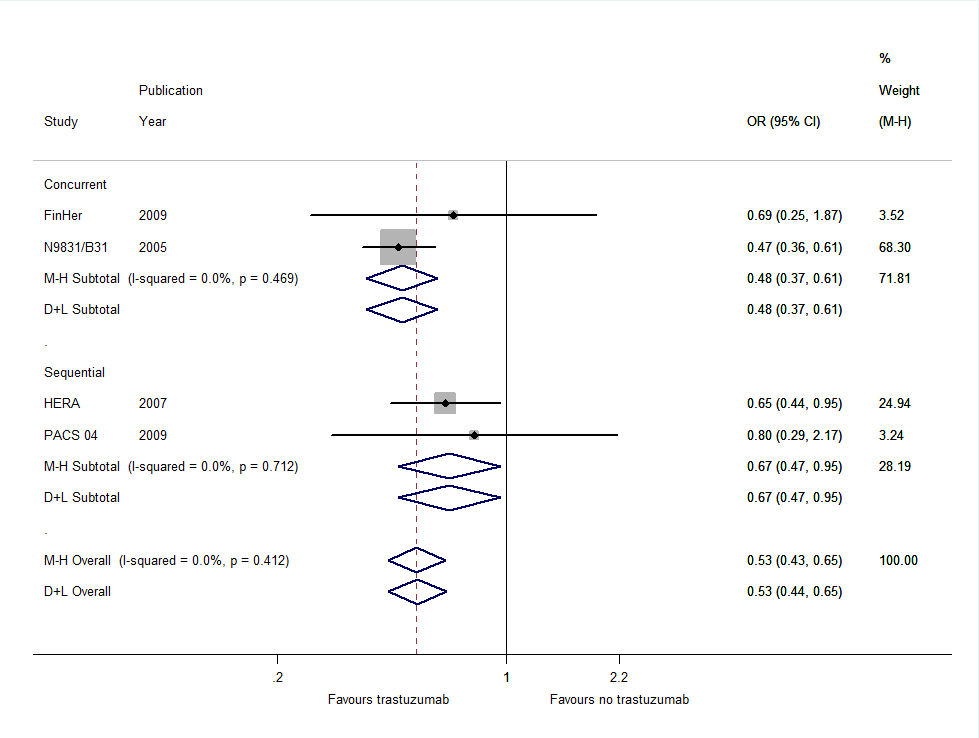

Supplement: Figure S4 — Forest plot of OR for the association between trastuzumab administration and locoregional recurrence by the timing of trastuzumab initiation with respect to chemotherapy. The size of the square box is proportional to the weight that each study contributes in the meta-analysis. The overall estimate and confidence interval are marked by a diamond. Symbols on the right of the solid line indicate OR>1 and symbols on the left of the solid line indicate OR<1. Abbreviations: M-H = Mantel-Haenszel (fixed-effects method); D+L = DerSimonian and Laird (random-effects method). (TIF) [file pone.0021030.s004.tif]

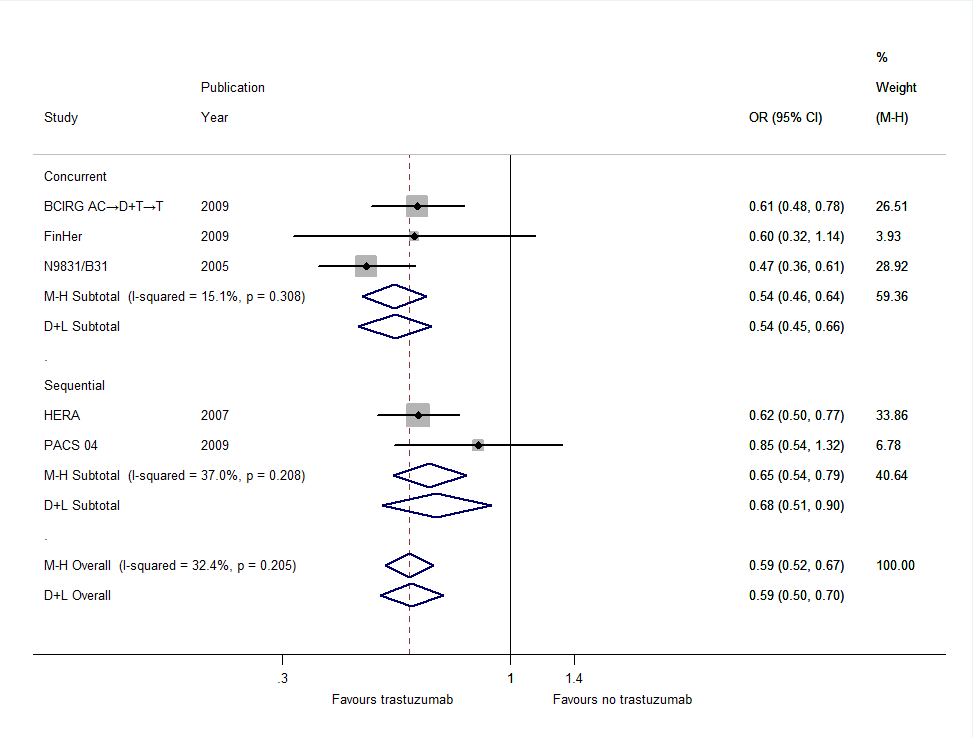

Supplement: Figure S5 — Forest plots of OR for the association between trastuzumab administration and distant recurrence by the timing of trastuzumab initiation with respect to chemotherapy without including the DCarboT arm of the BCIRG 006 trial. The size of the square box is proportional to the weight that each study contributes in the meta-analysis. The overall estimate and confidence interval are marked by a diamond. Symbols on the right of the solid line indicate OR>1 and symbols on the left of the solid line indicate OR<1. Abbreviations: M-H = Mantel-Haenszel (fixed-effects method); D+L = DerSimonian and Laird (random-effects method). (TIF) [file pone.0021030.s005.tif]

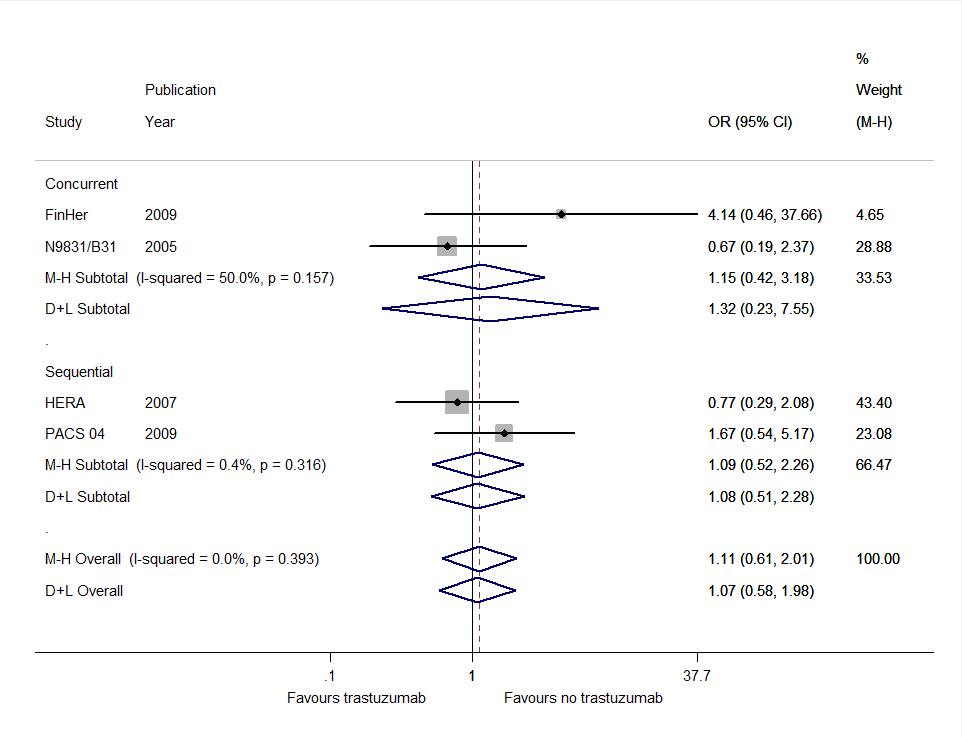

Supplement: Figure S6 — Forest plot of OR for the association between trastuzumab administration and contralateral breast cancer by the timing of trastuzumab initiation with respect to chemotherapy. The size of the square box is proportional to the weight that each study contributes in the meta-analysis. The overall estimate and confidence interval are marked by a diamond. Symbols on the right of the solid line indicate OR>1 and symbols on the left of the solid line indicate OR<1. Abbreviations: M-H = Mantel-Haenszel (fixed-effects method); D+L = DerSimonian and Laird (random-effects method). (TIF) [file pone.0021030.s006.tif]

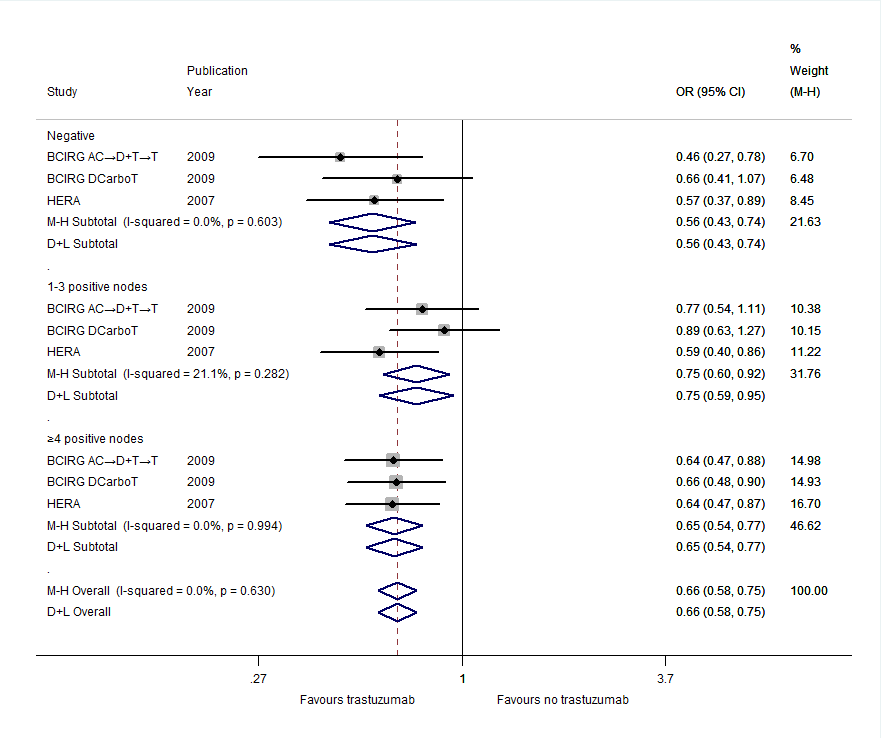

Supplement: Figure S7 — Forest plots of OR for the association between trastuzumab administration and DFS by axillary lymph node status (including the DCarboT arm of the BCIRG 006 trial). The size of the square box is proportional to the weight that each study contributes in the meta-analysis. The overall estimate and confidence interval are marked by a diamond. Symbols on the right of the solid line indicate OR>1 and symbols on the left of the solid line indicate OR<1. Abbreviations: M-H = Mantel-Haenszel (fixed-effects method); D+L = DerSimonian and Laird (random-effects method). (TIF) [file pone.0021030.s007.tif]

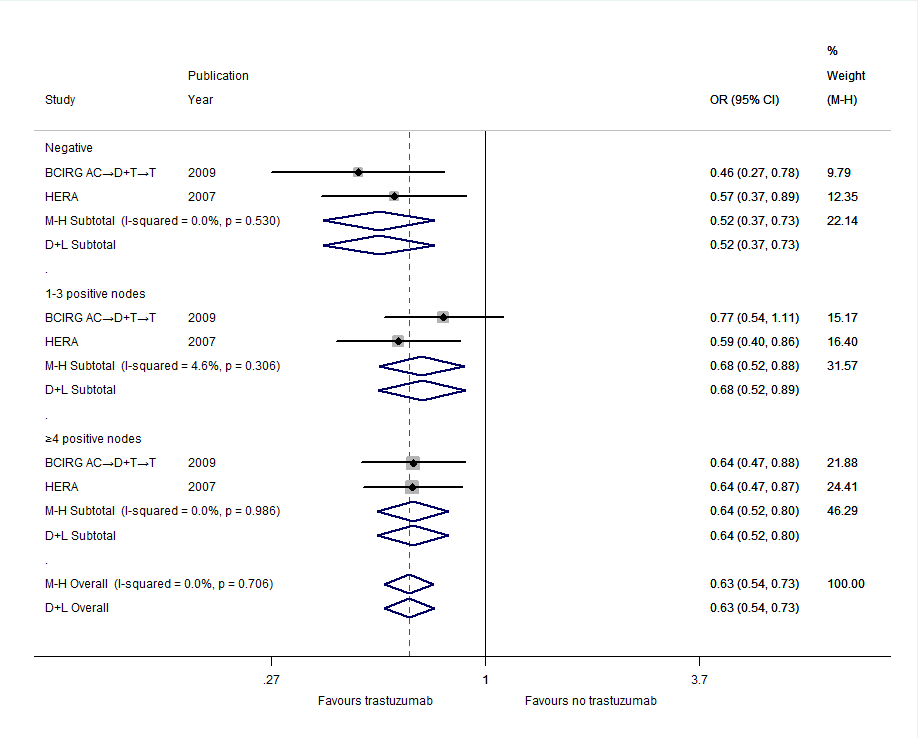

Supplement: Figure S8 — Forest plots of OR for the association between trastuzumab administration and DFS by axillary lymph node status without including the DCarboT arm of the BCIRG 006 trial. The size of the square box is proportional to the weight that each study contributes in the meta-analysis. The overall estimate and confidence interval are marked by a diamond. Symbols on the right of the solid line indicate OR>1 and symbols on the left of the solid line indicate OR<1. Abbreviations: M-H = Mantel-Haenszel (fixed-effects method); D+L = DerSimonian and Laird (random-effects method). (TIF) [file pone.0021030.s008.tif]

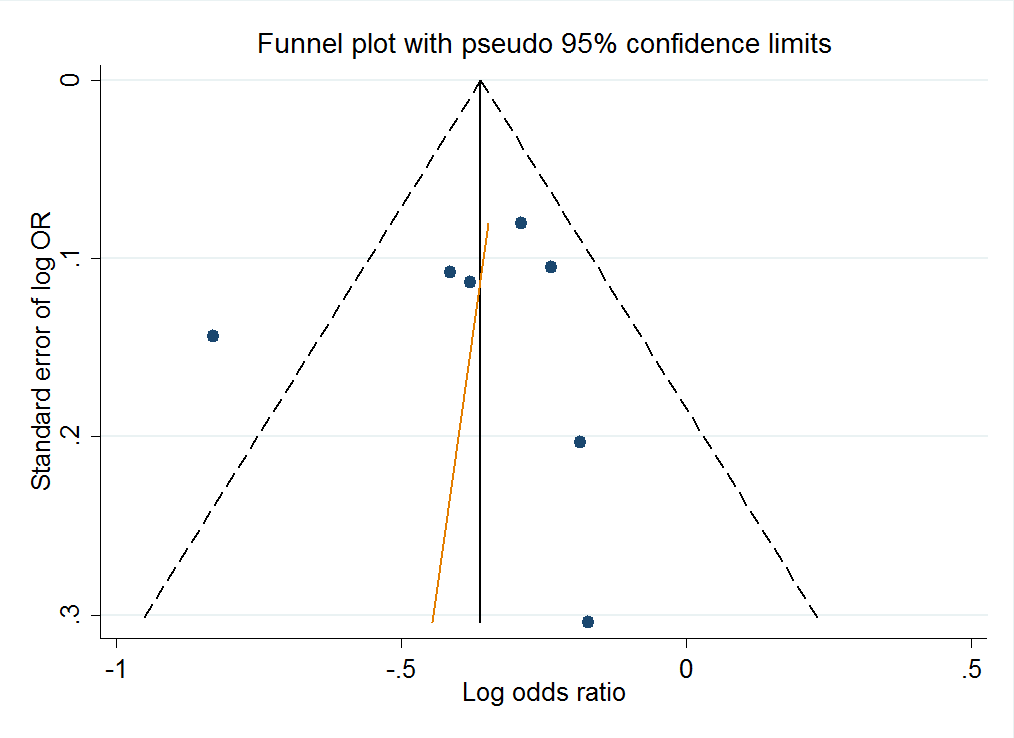

Supplement: Figure S9 — Funnel plot for publication bias in DFS with the analysis including the DCarboT arm of the BCIRG 006 trial. The red line indicates the fitted line corresponding to the Egger's regression test for funnel plot asymmetry. (TIF) [file pone.0021030.s009.tif]
