# Supplementary material for: Trastuzumab in the Adjuvant Treatment of HER2-Positive Early Breast Cancer Patients: A Meta-Analysis of Published Randomized Controlled Trials
Source: PLoS One. 2011 Jun 9;6(6):e21030. doi: 10.1371/journal.pone.0021030 (PMC3111470; doi:10.1371/journal.pone.0021030)
Supplement: Table S1 — Effect of trastuzumab in subgroup by the timing of trastuzumab initiation with respect to adjuvant chemotherapy. (DOC) [file pone.0021030.s011.doc]

**Table S1.** Effect of trastuzumab in subgroup by the timing of trastuzumab initiation with respect to adjuvant chemotherapy.

1. Concurrent group

|  | Heterogeneity test | | | |  | Significance test of OR=1 | |
| --- | --- | --- | --- | --- | --- | --- | --- |
| Variable | Heterogeneity chi-squared (d.f.) | I-squared | p | |  | OR (95% CI) | p |
| **DFS** |  |  | |  |  |  |  |
| DCarboH arm of the BCIRG 006 trial included | 11.93 (3) | 74.80% | | 0.01 |  | 0.645 (0.489-0.853) | <0.001 |
| DCarboH arm of the BCIRG 006 trial excluded | 6.92 (2) | 71.10% | | 0.03 |  | 0.593 (0.419-0.841) | <0.001 |
| **Overall survival** | 1.61 (2) | 0.00% | | 0.45 |  | 0.692 (0.576-0.833) | <0.001 |
| **Locoregional recurrence** | 0.52 (1) | 0.00% | | 0.47 |  | 0.480 (0.375-0.614) | <0.001 |
| **Distant recurrence** |  |  | |  |  |  |  |
| DCarboH arm of the BCIRG 006 trial included | 6.22 (3) | 51.70% | | 0.10 |  | 0.599 (0.522-0.688) | <0.001 |
| DCarboH arm of the BCIRG 006 trial excluded | 2.36 (2) | 15.10% | | 0.31 |  | 0.543 (0.458-0.643) | <0.001 |
| **Contralateral breast cancer** | 2.00 (1) | 50.00% | | 0.16 |  | 1.150 (0.416-3.181) | 0.787 |
| **CNS recurrence** | 0.41 (1) | 0.00% | | 0.52 |  | 2.240 (1.211-4.142) | 0.010 |

1. Sequential group

|  | Heterogeneity test | | |  | Significance test of OR=1 | |
| --- | --- | --- | --- | --- | --- | --- |
| Variable | Heterogeneity chi-squared (d.f.) | I-squared | p |  | OR (95% CI) | p |
| **DFS** |  |  |  |  |  |  |
| DCarboH arm of the BCIRG 006 trial included | 0.80 (2) | 0.00% | 0.669 |  | 0.736 (0.651-0.832) | <0.001 |
| DCarboH arm of the BCIRG 006 trial excluded | 0.80 (2) | 0.00% | 0.669 |  | 0.736 (0.651-0.832) | <0.001 |
| **Overall survival** | 1.57 (2) | 0.00% | 0.455 |  | 0.860 (0.731-1.012) | 0.069 |
| **Locoregional recurrence** | 0.14 (1) | 0.00% | 0.712 |  | 0.667 (0.467-0.954) | 0.027 |
| **Distant recurrence** |  |  |  |  |  |  |
| DCarboH arm of the BCIRG 006 trial included | 1.59 (1) | 37.00% | 0.208 |  | 0.655 (0.539-0.795) | <0.001 |
| DCarboH arm of the BCIRG 006 trial excluded | 1.59 (1) | 37.00% | 0.208 |  | 0.655 (0.539-0.795) | <0.001 |
| **Contralateral breast cancer** | 1.00 (1) | 0.40% | 0.316 |  | 1.085 (0.522-2.258) | 0.827 |
| **CNS recurrence** | 0.12 (1) | 0.00% | 0.725 |  | 1.247 (0.767-2.028) | 0.374 |

1. Overall

|  | Heterogeneity test | | |  | Significance test of OR=1 | |
| --- | --- | --- | --- | --- | --- | --- |
| Variable | Heterogeneity chi-squared (d.f.) | I-squared | p |  | OR (95% CI) | p |
| **DFS** |  |  |  |  |  |  |
| DCarboH arm of the BCIRG 006 trial included | 14.32 (6) | 58.10% | 0.026 |  | 0.687 (0.593-0.797) | <0.001 |
| DCarboH arm of the BCIRG 006 trial excluded | 12.59 (5) | 60.30% | 0.027 |  | 0.667 (0.562-0.792) | <0.001 |
| **Overall survival** | 6.13 (5) | 18.50% | 0.293 |  | 0.782 (0.692-0.883) | <0.001 |
| **Locoregional recurrence** | 2.87 (3) | 0.00% | 0.412 |  | 0.533 (0.435-0.652) | <0.001 |
| **Distant recurrence** |  |  |  |  |  |  |
| DCarboH arm of the BCIRG 006 trial included | 8.30 (5) | 39.70% | 0.141 |  | 0.617 (0.552-0.690) | <0.001 |
| DCarboH arm of the BCIRG 006 trial excluded | 5.92 (4) | 32.40% | 0.205 |  | 0.588 (0.518-0.668) | <0.001 |
| **Contralateral breast cancer** | 2.99 (3) | 0.00% | 0.393 |  | 1.107 (0.611-2.006) | 0.737 |
| **CNS recurrence** | 2.61 (3) | 0.00% | 0.456 |  | 1.578 (1.082-2.302) | 0.018 |

Abbreviations: d.f., degrees of freedom; OR, odds ratio; CI, confidence interval; DFS, disease-free survival; D, docetaxel; Carbo, carboplatin; H, trastuzumab; BCIRG, Breast Cancer International Research Group; CNS, central nervous system.
